# Analysis of the Antibacterial Activity and the Total Phenolic and Flavonoid Contents of the *Moringa oleifera* Leaf Extract as an Antimicrobial Agent against *Pseudomonas aeruginosa*

**DOI:** 10.1155/2023/5782063

**Published:** 2023-09-30

**Authors:** Ahmad Royani, Muhammad Hanafi, Puspa Dewi N. Lotulung, Heddy Julistiono, Achmad Dinoto, Azwar Manaf

**Affiliations:** ^1^Postgraduate Program of Materials Science Study, Department of Physics, Faculty of Mathematics and Natural Sciences, Universitas Indonesia, Depok 16424, Indonesia; ^2^Research Center for Metallurgy, National Research and Innovation Agency, Kawasan Puspiptek, Tangerang Selatan 15314, Indonesia; ^3^Research Center for Pharmaceutical Ingredients and Traditional Medicine, National Research and Innovation Agency (BRIN), Tangerang Selatan-Banten 15314, Indonesia; ^4^Research Center of Applied Microbiology, National Research and Innovation Agency, Cibinong Science Center, Cibinong 16911, Indonesia

## Abstract

*Pseudomonas aeruginosa* is a bacterium that causes metal deterioration by forming biofilms on metal surfaces. This work was carried out to analyze the antibacterial activity and the phenolic and flavonoid contents of the *Moringa oleifera* leaf extract against *Pseudomonas aeruginosa*. *M. oleifera* leaves were extracted in a methanol solution at different concentrations. The *M. oleifera* leaf extract yields were 12.84%, 18.96%, and 19.64% for the 100%, 75%, and 50% methanol ratios, respectively. Extracts of *M. oleifera* leaves had a minimum inhibiting concentration (MIC) of approximately 6144 *μ*g/mL against *P. aeruginosa* for a ratio of 100% methanol. In addition, no antibacterial activity was found for the 75% and 50% methanol ratios. The total phenolic levels were 16.26%, 12.73%, and 12.33% for the 100%, 75%, and 50% methanol solvent ratios, respectively. The total amounts of flavonoids were 23.32%, 3.40%, and 0.64% for the 100%, 75%, and 50% methanol solvents, respectively. The chemical structure of *M. oleifera* consists of kaemferol-3-O-rutinoside, quercimeritrin, kaempferol-3-O-*β*-D-glucopyranoside, stearidonic acid, trichosanic acid, pyrophaeophorbide A, and stigmastan-3,6-dione. The concentration of the solvent is essential in the extraction of plant constituents. Different concentrations indicate differences in antibacterial activity, phenolic and flavonoid contents, and chemical structure.

## 1. Introduction

The main bacterium in the marine environment is *Pseudomonas aeruginosa*, which can produce a biofilm layer on metal surfaces [1]. The reaction between the surface metal and biofilm layer and the differential aeration cell formation on the metal surface generate conditions that initiate and accelerate the corrosion rate [2]. This type of bacteria in biofilms can cause severe corrosion damage to steel [1] and other metals [3–5]. Microbiologically affected corrosion is a form of destructive corrosion that is started, aided, and facilitated by the presence of microbe activities [6, 7] and most generally manifests in holes localized on the surface material [8]. Bacteria attach to the substrate and form a biofilm layer, creating conditions that promote metal corrosion [9]. The bacteria inside the biofilm accelerate and create differential oxygen, resulting in corrosion and severe material damage [10]. Therefore, a comprehensive strategy is needed to overcome these biofilm-forming bacteria.

The creation of various biofilm layers on the metal substrate by bacteria results in the formation of differential aeration cells, which causes local corrosion [11]. The cathode is above the steel substrate and enriched with oxygen, while the anode is underneath a biofilm layer and lacks oxygen. The difference in the oxygen concentration between the anode and the cathode activates the electrochemical cells, resulting in localized corrosion in the form of pitting or crevices [12]. *P. aeruginosa* strains have also been linked to the oxidation of iron to ferrous iron (Fe^2+^), which exposes the steel to further dissolution because iron ions are more soluble than iron. This process attacked the protective layer of the steel surface [13].

Generally, the major bacterial corrosion mechanism has been classified into the following three phases [14]: (a) differential aeration cells caused by the formation of biofilms, which cause corrosion damage; (b) a reaction between a steel substrate and exopolysaccharide polymer substrate (EPS), in which EPS metal biominerals act as corrosion inducers; and (c) the role of siderophore bacteria in iron reduction. Corrosion control techniques due to bacteria are carried out physically and chemically; physically through regular cleaning of mucus and deposits in pipes [15], while chemically using biocides (chemical reagents) [16, 17]. The limit for chemical biocides is that they are toxic and ecologically unfriendly [18]. Recently, researchers have developed an ecofriendly biocide to address this issue [19–21].

Plants are a rich source of many active compounds, primarily secondary metabolites with antibiofilm, antifungal, and antibacterial functions [21]. Researchers have reported that some extraction plants are antimicrobial active and are used to control corrosion [22–24]. *Moringa oleifera* extracts have been studied for mild steel corrosion as a potential corrosion inhibitor in acidic environments [25]. The *M. oleifera* extract may also act as a resistance modifier, increasing the effectiveness of various antibiotics against certain bacteria [26]. As a result, the *M. oleifera* extract has the potential to inhibit bacterial growth in various environments.

Active compounds such as flavonoids and phenols have been identified to help inhibit the formation of biofilms [27, 28]. Therefore, an approach to inhibit the formation of biofilms involves identifying or extracting active compounds that act as inhibitors of biofilms. Maceration is one of the simple and inexpensive techniques for extracting the active substance from plants [29]. In addition, maceration is recommended as an extraction method with different solvents to extract high-quality antioxidant raw materials from the *M. oleifera* leaf to obtain a total phenolic and a maximum of flavonoids [30]. The *M. oleifera* methanol extract shows more antioxidant and antibacterial activities against foodborne pathogens than diethyl ether extracts [31]. These phenolic and flavonoid compounds have the potential to disrupt the bacterial cellular system by inactivating their receptors in the bacterial signaling pathway [27].

This study focused on extracting phenolic and flavonoid compounds and their antibacterial activities against *P. aeruginosa*. *M. oleifera* plants were extracted at room temperature in various methanol concentration ratios. The flavonoid and phenolic contents in the extract were analyzed by the colorimetric aluminum chloride and Folin–Ciocalteu methods. In addition, extracts were assessed for their suitability as antimicrobials to inhibit *P. aeruginosa*. *M. oleifera* extracts were also evaluated for their polarity structure and distribution with liquid chromatography-mass spectrometry (LC-MS/MS) and thin layer chromatography (TLC). To the best of our knowledge, no *M. oleifera* extract has been reported as a microbial corrosion or biocide inhibitor against biofilm-forming bacteria. Thus, this study is the first time as new green biocidal materials for antibiocorrosion additives would constitute new information. The results of this research are expected to serve as initial guidelines before extracts are used as antibiofilms and biocorrosion.

## 2. Materials and Methods

### 2.1. Reagents and Chemicals

The reagents and chemicals used in this work are methanol and distilled water as solvents for *M. oleifera* leaf extraction. Folin–Ciocalteu reagents, gallic acid, sodium hydroxide, anhydrous sodium carbonate, quercetin reagents, and anhydrous aluminum chloride are used to analyze the total phenolic and flavonoid contents. Meanwhile, nutrition (Brain Heart Infusion, BD Bacto), artificial seawater (Marine Art SF-1), MTT reagent (Thiazolyl Blue Tetrazolium Bromide), and propan-2-ol are used for assessing the bacterial activity.

### 2.2. Sampling and Identification of Plants

Samples of *M. oleifera* leaf were obtained from the Indonesian Medical and Aromatic Crops Research Institute (IMACRI), Bogor, West Java. These samples were identified in the Botany Laboratory (Herbarium Bogoriense), Directorate of Scientific Collection Management, National Research and Innovation Agency (BRIN).

### 2.3. Preparation of the Extract

The leaves of *M. oleifera* were carefully cleaned with running water to eliminate soil particles and dust. Then, this *M. oleifera* leaf was dried under the scorching sun for three days. *M. oleifera* dried leaf was ground to a size of <60 mesh. 25 g of *M. oleifera* powdered leaf was added to 150 mL of solvent with ratio concentrations of 100%, 75%, and 50% (*v*/*v*) methanol water, respectively. The extraction technique was performed by macerating at room temperature for 3 × 24 hours, and a new solution was replaced every 24 hours. After maceration, filtering is carried out and then concentrated in a rotating evaporator at 50°C. Finally, the *M. oleifera* leaf extract was refrigerated until further processing. The result of the extraction is determined according to the following formula:

(1) $$\begin{array}{c} \text{Yield }\left(\%\right)=\left(\frac{W_{1}}{W_{0}}\right)\times 100, \end{array}$$

where *W*_1_ represents the final weight of the *M. oleifera* leaf extract (concentrated extract) and while *W*_0_ represents the initial weight of *M. oleifera* dried leaf (*M. oleifera* leaf powder).

### 2.4. Thin Layer Chromatography (TLC) Analysis

The TLC test was observed to analyze the polarity distribution of the active components contained in the *M. oleifera* leaf extract at various methanol ratios. Identification was performed using silica gel as a stationary phase and various *n*-hexane to ethyl acetate in a ratio of 1 : 0 to 0 : 1 as a mobile phase. Detection of chromatograms was observed with or without ultraviolet (UV) light. The UV light observations were made at 254 nm and 366 nm wavelengths.

### 2.5. Strains and Cultures of Bacteria

*Pseudomonas aeruginosa* strain from Indonesian Culture Collection (InaCC) B3 was utilized in the antibacterial research assay. This bacterial strain was obtained from BRIN under the license of InaCC. The oblique bacteria culture was moved to the freshwater brain heart infusion (BHI) broth media and then transferred to the marine BHI broth media as a test material.

### 2.6. Antibacterial Test

The antibacterial activity and minimum inhibitory concentration (MIC) were assessed using the MTT methods [32, 33]. The media was prepared in accordance with the manufacturing instructions. 37 g of BHI were suspended in 1000 mL of distilled water and then mixed, heated, and boiled for 1 minute until fully dissolved. It is then autoclaved at 121°C for about 15 minutes. Furthermore, artificial seawater was prepared with the following manufacturing instructions: 38 g of powders (Marine Art SF-1) were dissolved into 1000 mL of distilled water, followed by heating and mixing. Subsequently, the solution was autoclaved for 15 minutes at 121°C.

In summary, an aliquot of 100 *μ*L (BHI-artificial sea water in a ratio of 1 : 1) consisting of different concentrations of the extract was added to each plate of 96 wells. The extract concentration varied from 0 *μ*g/mL and 512 *μ*g/mL up to 6144 *μ*g/mL for this study. Each tube well is filled with a suspension of bacteria cells (2 *μ*L) from a 24-hour culture. Microplates are incubated for 24 hours at room temperature. Furthermore, 10 *μ*L of MTT solution (containing 5 mg/mL) was filled into the tube well and incubated for 1 hour. The well was then filled with 10 *μ*L of MTT solution (5 mg/mL) and incubated for 1 hour. Afterwards, each tube well was filled with 100 *μ*L of propan-2-ol containing 0.04 M of HCl. A microplate reader measured cell suspension absorption at 595 nm (Bio-Rad xMark). All experiments were carried out in three replications. The percentage of inhibition against viable cells was determined using the following equation [30]:

(2) $$\begin{array}{c} \text{Percentage inibition }\left(\%\right)=\left[1-\left(\frac{{\text{Abs}}_{t}}{{\text{Ass}}_{c}}\right)\right]x\;100, \end{array}$$

where Abs_*t*_ and Abs_*c*_ are cell absorbance treated and cell absorbance control, respectively.

### 2.7. Total Phenolic and Flavonoid Analysis

The total phenolic compounds in the extract were estimated to follow the previously reported [34, 35] using Folin–Ciocalteu methods with gallic acid as reference. The sample solution and the reference solution of gallic acid are each placed into a test tube and then dried into 4 mL by adding aquades, Folin–Ciocalteau 250 *μ*L, and shaken. After 8 minutes, 750 *μ*L of 20% Na_2_CO_3_ was added and shaken homogeneously. This mixture is then left for 2 hours at room temperature. Absorption was read by using a spectrophotometer at 765 nm.

The total flavonoid compounds were analyzed by the colorimetric aluminum chloride method [36] with quercetin as a standard solution. 4 mg of each quercetin was used, and the extracted sample was dissolved in 4 ml of methanol. Pipetting up to 250 *μ*L of sample into the test tube allowed for accurate measurements. 2 ml of aquades and 150 *μ*L of 5% NaNO_2_ were added to each test tube. 150 *μ*L of 10% AlCl_3_ was added after 5 minutes. Six minutes later, 2 ml of NaOH 1 M was added, and the volume was calibrated to 5 mL with the addition of aquades. The mixture was homogenized and measured using a UV-Vis spectrophotometer at 510 nm.

### 2.8. Analysis of Structural Compounds

The shape and active compounds in the *M. oleifera* leaf extract were determined using structural analysis. LC/MS-MS was used to examine the compound structure of the *M. oleifera* extract (Agilent Technologies 7890). For 17 minutes, structural compounds were measured using LC-MS-MS at 0.3 mL/min of flow rate.

## 3. Results and Discussion

**Figure 1:**
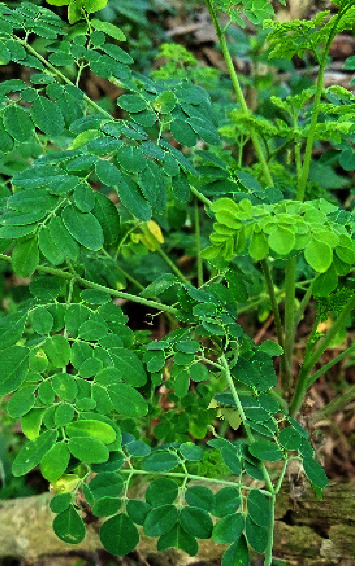
*M. oleifera* leaf was used in this work.

Samples of *M. oleifera* leaves (Figure 1) used in this study have been identified at the Botanical Laboratory (Herbarium Bogoriense) under the Directorate of Scientific Collection Management, National Research and Innovation Agency, BRIN, with certificate number B-1809/II.6.2/DI.05.07/6/2022. The following are the results of determining the *M. oleifera* plant used in this work, as tabulated in Table 1.

### 3.1. Yield Extracts

The yield of *M. oleifera* leaf (25 g) with various methanol solvent ratios was 12.84, 18.96%, and 19.64% for 100%, 75%, and 50%, respectively (Table 2). According to Table 2, solvents with a higher polarity (i.e., a larger ratio of water solvents) extract more significant quantities. Conversely, extracts with a lower solvent polarity (100% methanol) also have a lower percentage of the extract. Fewer polar compounds can be extracted with a methanol ratio of 100%. In the maceration of the plant, the breakdown of cell walls and membranes was caused by pressure differences, so the secondary metabolites in the cytoplasm will be dissolved in the organic solvent [37]. In addition, the maceration process can be carried out without heat, providing that the secondary compounds to be observed were not damaged [38].

These results are consistent with the recent report, which demonstrated that aqueous solvents produce extracts higher than methanol solvents [39]. Other researchers reported that extracting *M. oleifera* leaves (1.5 g) with different polarity solvents resulted in dry extracts with a weight of 5%–36% (*M. oleifera* leaf hexane extract) [26]. Hexane, ethyl acetate, and chloroform extracts generally produced a relatively low mass of extracted material compared to water and methanol extracts [27]. Hence, the type of solvent is critical in extracting the active content from the plant [28].

The use of methanolic solvents in maceration was based on the fact that methanolic has a boiling point of 65°C and a polarity index of 5.1. In comparison, ethanol has a boiling point of 78°C and a polarity index of 4.9 [40]. Therefore, the evaporation process uses a rotary evaporator to obtain a viscous extract using methanol faster than ethanol. The temperature for evaporating the sample with methanolic solvent is not very high, minimizing the risk of overheating and destroying the secondary metabolite content in the sample. Consequently, the processing time needed to evaporate the sample is relatively quick. Furthermore, extraction studies using various solvents revealed that methanolic is the best solvent for extracting bioactive contents with the highest extract yield [41].

### 3.2. Thin Layer Chromatography (TLC)

**Figure 2:**
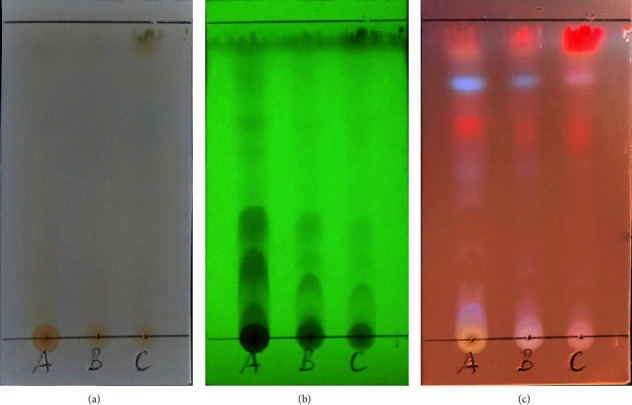
The chromatogram pattern of the crude extract of *M. oleifera* for methanol concentration ratios (A (50% MeOH), B (75% MeOH), and C (100% MeOH)) at (a) non-UV light, (b) 254 nm, and (c) 366 nm.

TLC identification results showed polarity behavior with an increase in the ratios of ethyl acetate at the ratios of extracts of 100% methanol and methanol 75% and 50%. This difference is due to the higher polarity of ethyl acetate solutions with respect to *n*-hexane solutions [40]. The chromatogram pattern resulting from the extract on the TLC plate was examined under visible light and UV light at 366 nm and 254 nm of wavelengths, as shown in Figure 2. Figure 2 shows the polarity spot distribution of the active metabolite in the *M. oleifera* leaf extract and the ratio effect of methanol solvents in the variation of n-hexane: ethyl acetate as the mobile phase.

The value of the retention factor, *R*_*f*_ (ratio of distance traveled by the substance to the solution), is an important parameter used for qualitative TLC analysis [42]. The two components are similar molecules if two points travel the same distance or have the same *R*_*f*_ value. In this study, qualitative tests for all the three extract ratios revealed similar indications of metabolite. TLC profiling of all the three extract ratios yielded impressive results, indicating the presence of several phytochemicals. In different solvent systems, different phytochemicals have different *R*_*f*_ values [43]. Pure compounds can be separated from plant extracts using different ratios of solvents with variable polarity. Only by analyzing the *R*_*f*_ value of the compound in different solvent systems can the correct solvent system for a specific plant extract be determined [43]. These findings will aid in selecting an appropriate solvent system for further compound separation from this plant extract.

### 3.3. Antibacterial Activity

**Figure 3:**
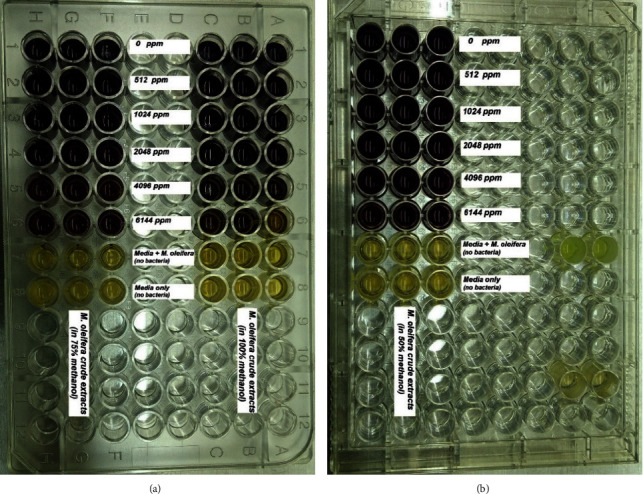
The activity of *P. aeruginosa* with various concentrations of *M. oleifera* using the microdilution method: (a) 75% and 100% methanol and (b) 50% methanol.

The results of the antibacterial activity test of the *M. oleifera* leaf extract with variations in concentration are shown in Figure 3. Analysis of optical density data obtained for all experiment repetitions showed the average OD value (± standard deviation) against the control.

The microdilution method determined the minimum inhibitory concentration (MIC) of the *M. oleifera* leaf extract. MIC is defined as the minimum concentration required to inhibit the bacterial growth. At a methanol concentration ratio of 100%, the initial inhibition of bacteria is indicated by a brighter color than other concentrations (75% and 50% of the methanol solvent) in the 6144 *μ*g/mL concentration extract. At the same time, the *M. oleifera* leaf extract could not inhibit the growth of *P. aeruginosa* (dark blue color) at a concentration of 75% like at a methanol concentration of 50%. The indicator of minimum inhibition was observed by the degradation of the blue color of the MTT seawater to be lighter.

**Figure 4:**
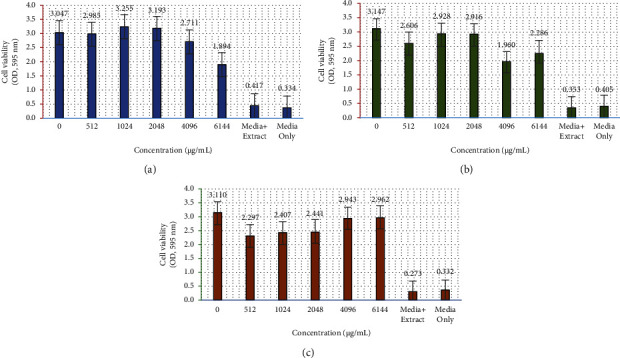
Cell viability versus concentration in various methanol extract ratios: (a) in 100% methanol, (b) in 75% methanol, and (c) in 50% methanol.

**Figure 5:**
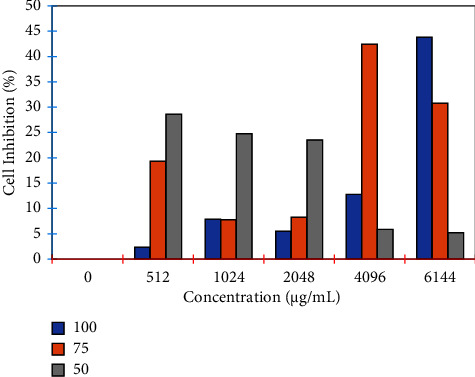
The percentage of inhibition of the *M. oleifera* leaf extract at various concentrations.

The test results confirmed that the *M. oleifera* leaf methanol extract has antibacterial activity against the bacteria tested (*P. aeruginosa*) with tabulated absorbance in the graph in Figure 4. The percentage of the antibacterial activity of *M. oleifera* leaf extract against *P. aeruginosa* is presented in the graph in Figure 5. At a ratio of 100%, a concentration of 6144 ug/mL of the *M. oleifera* leaf extract can inhibit the growth of *P. aeruginosa* by about 43.84%; at the same concentration (6144 ug/mL), *M. oleifera* extracts only inhibit the growth of *P. aeruginosa* by 27.36% and 4.76% for the ratios of 75% and 50%, respectively.

The ability of active content from the *M. oleifera* leaf extract against *P. aeruginosa* was investigated in this study. The antibacterial activity tests show that the *M. oleifera* leaf extract can inhibit bacterial growth, although it is still relatively low. Another report found that the *M. oleifera* leaf extract had varying antimicrobial activity in various microorganisms [44]. Bacterial growth inhibition based on extract concentrations implies that increasing the concentration of the methanol extract increases inhibitory absorption. These findings support previous research that *M. oleifera* leaf powder has antibacterial activity against negative bacteria at a low level [39]. Kumar et al. [45] reported that the *M. oleifera* extract with the dilution method has an antibacterial activity with a minimum inhibitory concentration of 7.4 mg/mL and 2.4 mg/mL for *S. aureus* and *E. coli*, respectively.

Another researcher confirmed that the *M. oleifera* crude extract had no inhibition against *P. aeruginosa* ATCC 27853 at 175 *μ*g/mL of concentration in the purification and characterization of phytocystatin isolated from *M*. *oleifera* [46]. In addition, plants in various solvents showed different activities against *P. aeruginosa* biofilms [28]. Another work found that the efficacy of 10 mL of the *M. oleifera* seed extract can cause Gram-positive (*B. subtilis*) and Gram-negative (*E. coli*) bacterial decay to a maximum of 93.2% and 96.2%, respectively [47]. The inhibitory activity of plant extracts against bacteria varies depending on the type of plant extracted [48, 49], the method and solvent used [49, 50], the type of target bacteria [39], and the content of active compounds in the extract [27].

The effectiveness of this inhibition against bacteria was caused by bioactive contents in the extract, which can damage cell walls (DNA) so that their growth slows down and even causes bacterial death [50]. The inhibition of DNA replication will cause bacteria to be unable to divide themselves, thus inhibiting the growth of bacteria. Other sources stated that flavonoids are one of the essential secondary metabolites identified as potential antimicrobial agents against various pathogenic microorganisms [51]. Flavonoids have an antibacterial effect due to their many biological actions, which may seem not very specific initially. However, prospective antibacterial flavonoids effectively target bacterial cells and inhibit virulence factors and other types of bacterial risks, such as biofilm formation.

### 3.4. Total Phenolic and Flavonoid Contents

The effect of the ratio of methanol to the bioactive contents of the *M. oleifera* leaf extract is presented in Table 3. This study revealed a considerable difference in the percentage of bioactive compounds (flavonoids) to the ratio of methanol solvents in *M. oleifera* extracts.

The resulting equation of the fault acid reference curve is *y* = 0.1032*x* + 0.0708; *r*^2^ = 0.9996. The total values of the phenolic content (TPC) are about 16.26%, 12.73%, and 12.33% for methanol ratios of 100%, 75%, and 50%, respectively, as described in Table 3. The total value of the percentage of the phenolic content decreases ramps with a decrease in the solvent ratio of methanol. This reduction may be due to methanol attracting polar and nonpolar compounds while water attracts only polar compounds. Our results aligned with Elboughdiri [52], which found that the total phenolic content (TPC) decreased rampantly when the solvent concentration ratio was lowered. The phenolic content also depends on the plant organs [53]. For example, a study of extracts on different parts of *M. oleifera* reported that the total phenolic content was greater in the leaves than in other organ parts (whole seeds, kernels, mantle, and pods) [54]. In addition, the phytochemical properties (phenolics and flavonoids) of the *M. oleifera* leaf extract were also significantly affected by leaf age [49].

Meanwhile, in the standard curve of quercetin, the linear equation is *y* = 0.0073*x* − 0.0802 with *r*^2^ = 0.995. According to the results, the TFC extract values were 23.32%, 3.40%, and 0.64% for methanol ratios of 100%, 75%, and 50%, respectively. The TFC value of the *M. oleifera* extract differs markedly by different methanol ratios. Biochemical studies of the *M. oleifera* leaf extract reported that flavonoids were obtained with methanol solvents [55]. In addition, the significance of secondary metabolites in inhibiting *P. aeruginosa* biofilms was revealed by a strong connection between the flavonoid concentration and antibiofilm activity in the methanol extract [28].

Based on the total content of phenolics and flavonoids, optimal conditions were obtained at a methanol ratio of 100% to extract bioactive components from the *M. oleifera* leaf. The highest total phenolic content (about 16.26%) and flavonoids (about 23.32%) were obtained in these conditions. At the same time, a 50% methanol solvent shows lower efficiency in extracting phenolic compounds and flavonoids by 12.33% and 0.64%, respectively. These results are confirmed by the data of the bacterial activity test results that the highest inhibition was obtained at a concentration ratio of 100% methanol (Figure 5).

### 3.5. The Structure of the *M. oleifera* Leaf Extract

**Figure 6:**
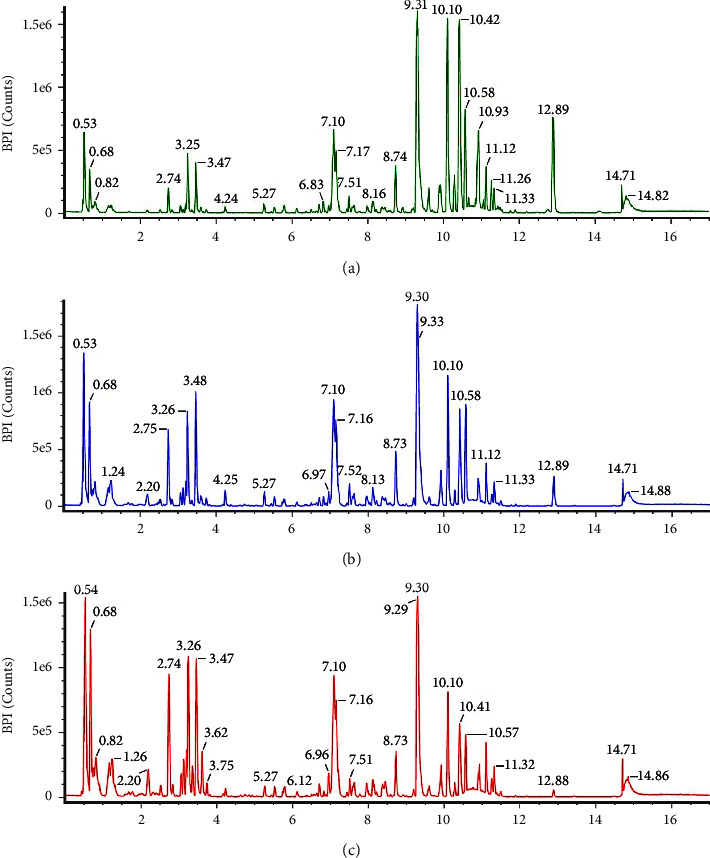
The result of the chromatogram of *M. oleifera* in various methanolic solvents: (a) in 100% methanol, (b) in 75% methanol, and (c) in 50% methanol.

The chromatogram results with LC-MS/MS are presented in Figure 6. These results illustrate the difference in the compound content of the methanol extract of *M. oleifera* leaf at different concentration ratios. This difference in active compounds is explained by the peak chromatograms of compounds of different molecular weights. The bioactive content of all methanol concentration ratios has the same compound, trichonic acid (C_18_H_30_O_2_), at a retention time of 9.31. The results of molecular weight analysis with LC-MS/MS showed that the active compound was found at methanol ratios of 100%, 75%, and 50%, as shown in Figure 6.

Figure 6 presents various compounds with varying degrees of peak intensity. In the compound at retention times 0.53, 0.68, 2.75, 3.26, 3.48, and 7.10, the peak intensity increases with a decrease in the methanol concentration ratio. In contrast, in the compound at a retention time of 9.31, 10.10, 10.42, and 12.89, peak intensities tend to get lower with a decrease in the concentration ratio of methanol solvents. The polarity of the extracted active compounds can cause differences in the intensity of such compounds. The compounds in the 50% methanol extract may be more polar than those in the 75% and 100% ratio. While these compounds are only found in 100% methanol, such compounds tend to have a lower polarity than compounds at their other methanol concentration ratios. This difference is because water solvents are more polar than methanol solvents [56]. The name and formula of the active content in the *M. oleifera* leaf extract are tabulated in Table 4.

**Figure 7:**
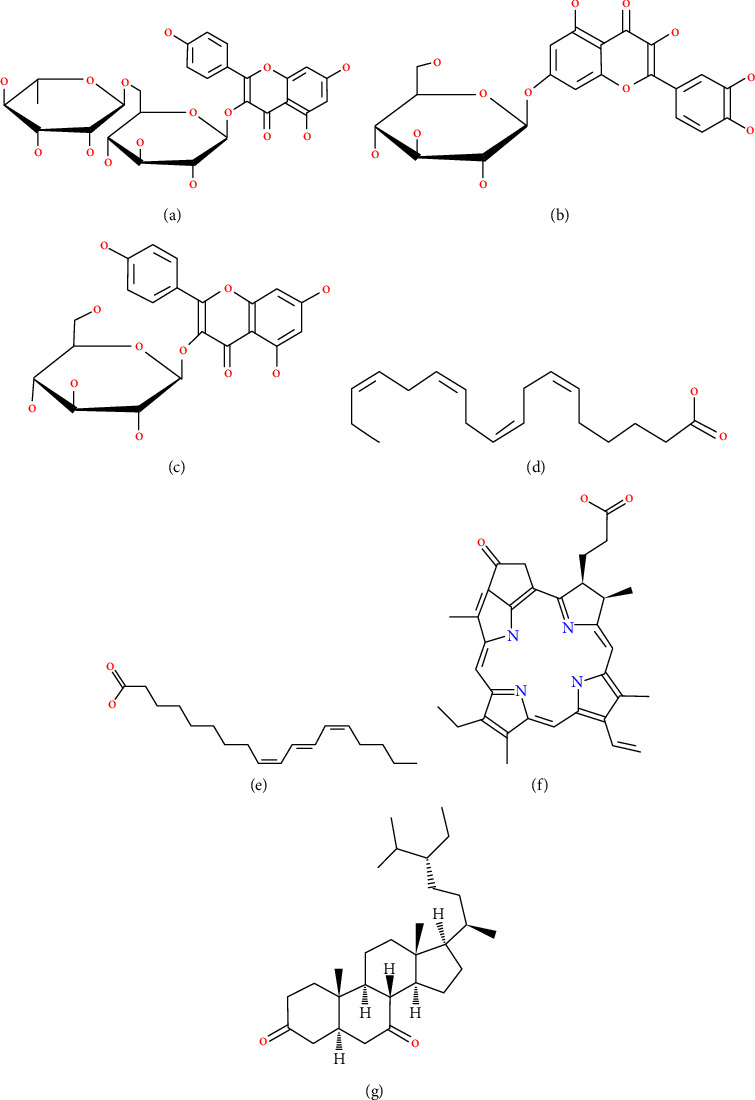
The structure of the active compounds in the extract of *M. oleifera* leaf: (a) kaempferol-3-O-rutinoside, (b) quercimeritrin, (c) kaempferol-3-O-*β*-D-glucopyranoside, (d) stearidonic acid, (e) trichonic acid, (f) pyrophaeophorbide A, and (g) stigmastan-3,6-dione.

The active structure can be obtained from a chromatogram at a specific retention time (RT) of different intensities (peak); for example, kaempferol-3-O-*β*-D-glucopyranoside (C_21_H_20_O_11_) and trichosanic acid (C_18_H_30_O_2_) at retention times of 3.48 and 9.31. Other compound structures resulting from the *M. oleifera* leaf extract are kaempferol-3-O-rutinoside (C_27_H_30_O_15_), suercimeritrin (C_21_H_20_O_12_), stearidonic Acid (C_18_H_28_O_2_), pyrophaeophorbide A (C_33_H_34_N_4_O_3_), and stigmastan-3,6-dione (C_29_H_48_O_2_), as shown in Figure 7.

The development of bioactive compounds in some plants can be used for various purposes, both in the medical field and in others. Due to the bioactive content in the *M. oleifera* plant, this plant has the potential for antimicrobial and antioxidant activity. *M. oleifera* consists of a large number of secondary metabolites [57]. Nizioł-Łukaszewska [59] concluded that the tested *M. oleifera* leaf extract contained high flavonoid and phenolic compounds and antioxidant potential and positively affected cell proliferation and metabolism at concentrations up to 5%. According to other sources, the phytochemicals of *M. oleifera* had a high amount of terpenoids, tannins, flavonoids, saponins, glycosides, alkaloids, and phenolic content [58]. In our study, the result of the structure of the extracted compound of *M. oleifera* is polyphenol glycoside (flavonoid glycoside), polyunsaturated fatty acid (PUFA), and alkaloid compounds, as shown in Figure 7. Flavonoids provide antibacterial activity by inhibiting nucleic acid production, cytoplasmic membrane function, energy metabolism, adhesion and biofilm development, suppression of porins in cell membranes, alterations in membrane permeability, and pathogenicity [60]. Meanwhile, the antibacterial activity of polyphenols mostly depends on their interaction with the bacterial cell surface [61].

## 4. Conclusions

Synergis investigations successfully assessed the antibacterial activity of *M. oleifera* leaf extract with various methanol ratios against *P. aeruginosa*. The extract yields were 12.84%, 18.96%, and 19.64% for 100%, 75%, and 50% methanol-water ratios, respectively. The total phenolic contents (TPCs) in the *M. oleifera* methanol extract were 16.26%, 12.73%, and 12.33% at 100%, 75%, and 50% methanol, respectively. At 100%, 75%, and 50% methanol, the TFCs were 23.32, 3.40, and 0.64%, respectively. The 100% methanolic *M. oleifera* leaf extract exhibited antibacterial activity against *P. aeruginosa*. The study demonstrated no bactericidal action at 75% and 50% methanolic concentrations. The minimum inhibitory concentration (MIC) of the *M. oleifera* leaf extract was 6144 g/mL with a 43.86% efficiency at a 100% methanolic ratio. The *M. oleifera* extract compounds included polyphenol glycoside (flavonoid glycoside), polyunsaturated fatty acid (PUFA), and alkaloid. Kaempferol-3-O-rutinoside, quercetin, kaempferol-3-O-D-glucopyranoside, stearidonic acid, trichosanic acid, pyrophaeophorbide A, and stigmastan-3,6-dione were identified as the active compounds in the *M. oleifera* leaf extract. The solvent concentration ratio is essential in extracting plant components. Various ratios indicate differences in the antibacterial activity, the total contents of phenolics and flavonoids, and the chemical structures of the compounds.

## Figures and Tables

**Table 1:** The determination results for the plant material used in this work.

No.	Classification of plant taxonomic	
1	Kingdom	Plantae
2	Division	Spermatophyta
3	Subdivision	Angiosperms
4	Class	Dicotyledoneae
5	Ordo	Brass
6	Families	Moringaceae
7	Genus	*Moringa*
8	Species	*Moringa oleifera Lam*.

**Table 2:** The yield of *M. oleifera* extracts in 3 different ratios of methanol solvents.

Ratio (methanol: H_2_O) (in *v*/*v*)	Weight of *M. oleifera* (grams)	Vol. solution (mL)	Extract yield of *M. oleifera* (%)
100 : 00	25	150	12.84
75 : 25	25	150	18.96
50 : 50	25	150	19.64

**Table 3:** The phenolic and flavonoid contents in the *M. oleifera* leaf extract in various methanolic solvents.

Plant extracts	Ratio (methanol: H_2_O)	Total phenolic content (%)	Total flavonoid content (%)
*M. oleifera*	100 : 00	16.26 ± 0.38	23.32 ± 0.63
	75 : 25	12.73 ± 0.70	3.40 ± 0.18
	50 : 50	12.33 ± 0.88	0.64 ± 0.03

**Table 4:** The name of the active content component in the *M. oleifera* leaf extract.

Plant extracts	Component name	Formula	Observed RT (min)	Neutral mass (Da)
*M. oleifera*	Kaempferol-3-O-rutinoside	C_27_H_30_O_15_	2.75	594.15847
	Quercimeritrin	C_21_H_20_O_12_	3.26	464.09548
	Kaempferol-3-O-*β*-D-glucopyranoside	C_21_H_20_O_11_	3.48	448.10056
	Stearidonic acid	C_18_H_28_O_2_	7.10	276.20893
	Trichonic acid	C_18_H_30_O_2_	9.31	278.22458
	Pyrophaeophorbide A	C_33_H_34_N_4_O_3_	10.42	534.26309
	Stigmastan-3,6-dione	C_29_H_48_O_2_	12.89	428.36543

## Data Availability

The datasets used or analyzed during the current study are included within the article and can be obtained from the corresponding author.
